# Optimal target blood pressure for the primary prevention of hemorrhagic stroke: a nationwide observational study

**DOI:** 10.3389/fneur.2023.1268542

**Published:** 2023-10-09

**Authors:** Hwan Seok Shim, Jeong-Mee Park, Yong Jae Lee, Young-Deok Kim, Tackeun Kim, Seung Pil Ban, Jae Seung Bang, O-Ki Kwon, Chang Wan Oh, Si Un Lee

**Affiliations:** Department of Neurosurgery, Seoul National University Bundang Hospital, Seoul National University College of Medicine, Seongnam-si, Republic of Korea

**Keywords:** blood pressure, stroke, intracerebral hemorrhage, subarachnoid hemorrhage, young adult

## Abstract

**Background:**

There are few reports on the preventative value of intensive blood pressure (BP) management for stroke, especially hemorrhagic stroke (HS), after new criteria for hypertension (HTN) were announced by the American College of Cardiology/American Heart Association in 2017.

**Aims:**

This study aimed to identify the optimal BP for the primary prevention of HS in a healthy population aged between 20 and 65 years.

**Methods:**

We conducted a 10-year observational study on the risk of HS, subclassified as intracerebral hemorrhage (ICH) and subarachnoid hemorrhage (SAH) according to BP categories (e.g., low normal BP, high normal BP, elevated BP, stage 1 HTN, and stage 2 HTN) using the National Health Insurance Service Database.

**Results:**

Out of 8,327,751 participants who underwent a health checkup in 2008, 949,550 were included in this study and observed from 2009 to 2018. The risk of ICH was significantly increased in men with stage 2 HTN {adjusted hazard ratio [aHR] 2.002 [95% confidence interval (CI) 1.203–3.332]} and in women with stage 1 HTN [aHR 2.021 (95% CI, 1.251–3.263)]. The risk of SAH was significantly increased in both men [aHR 1.637 (95% CI, 1.066–2.514)] and women [aHR 4.217 (95% CI, 2.648–6.715)] with stage 1 HTN. Additionally, the risk of HS was significantly increased in men with stage 2 HTN [aHR 3.034 (95% CI, 2.161–4.260)] and in women with stage 1 HTN [aHR 2.976 (95% CI, 2.222–3.986)].

**Conclusion:**

To prevent primary HS, including ICH and SAH, BP management is recommended for adults under the age of 65 years with stage 1 HTN.

## Introduction

The incidence and diagnosis rates of cerebrovascular diseases related to stroke are increasing as the population ages and diagnostic technology is developed (1–3). Accordingly, the demand for research on the primary or secondary prevention of stroke is also increasing. Among the modifiable risk factors for stroke, hypertension (HTN) is an important common factor in several studies (4). In 2017, the American College of Cardiology/American Heart Association (ACC/AHA) released an updated guideline with new criteria for HTN, defining stage 1 HTN as a systolic blood pressure (BP) of 130–139 mm Hg or a diastolic BP as 80–89 mm Hg (5).

However, although several studies have reported new diagnostic criteria for HTN that lower the incidence of various cardiovascular events (CVEs) (6–8), there are few helpful reports on the prevention of stroke. Some studies reported that intensive BP control prevents secondary stroke (3, 9, 10), but few studies reported a significant correlation with primary stroke prevention (8). Studies reporting that intensive BP control helps prevent primary stroke have limitations in that they include only patients with specific diseases, such as diabetes, or subgroup analysis, such as for hemorrhagic or ischemic stroke, was not conducted (9).

Therefore, we conducted a large-scale observational study on the risk of hemorrhagic stroke (HS), subclassified as subarachnoid hemorrhage (SAH) and intracerebral hemorrhage (ICH) according to BP based on the new diagnostic criteria for HTN in a healthy population using a nationwide cohort.

## Methods and materials

### Data source

We conducted a nationwide observational study using the National Health Claims Database, which is available from Korea's National Health Insurance Service (NHIS). The NHIS is a nationwide universal insurance system in Korea responsible for the nation's healthcare and medical bills. The database consists of hospital records, including admission and outpatient visit records, drug prescriptions, and national health examination data, for 97% of the Korean population. Adults in Korea are required to undergo national health examinations biannually, provided by the NHIS (11). The health examination includes measurements of height, weight, and BP, laboratory tests for urine and blood, and a questionnaire on medical history, family history, and health behaviors. The NHIS provides information from claim data for research purposes, and the NHIS database has been validated in several previous epidemiological studies (11). Using the International Classification of Diseases, 10th Revision (ICD-10) diagnostic codes, we collected data from patients with a primary outcome or medical history. The Seoul National University Bundang Hospital Institutional Review Board approved this study (IRB number: X-1910-572-903), and the requirement for informed consent was waived due to the retrospective nature of this study.

### Study population

Adults aged 20 years or older who underwent a national health examination in 2008 were included in this study, and patients aged 65 years or older were excluded to limit the number of participants who had missing values or who died from causes other than stroke to <5% during the observation period.

### Blood pressure measurement and participant selection

Among participants who were under 65 years of age, those who had missing values or who died from causes other than stroke during the observation period were subject to listwise deletion. Furthermore, participants who had a history of brain trauma (ICD-10 codes S06–S09) or brain tumor (ICD-10 codes C41.0, C75.2, C71, C79.3, D32.9–D333, D35.3, or D44.4) during the observation period or who had been diagnosed with brain trauma, brain tumor, or stroke (ICD-10 codes I60–I63) during the preceding 5-year period were excluded to extract only those who were diagnosed with stroke for the first time during the observation period and to minimize the effects of other causes on the brain. To increase the reliability of the medical data, we used data extracted exclusively from tertiary general referral hospitals, general hospitals, and semi-general hospitals.

BP was measured three times after participants rested for at least 2 min in a sitting position by digital or automatic monitors during the health examination, and mean BP was recorded. All participants were categorized by BP measurements: low normal (systolic, <110 mm Hg; diastolic, <80 mm Hg), high normal (systolic, 110–119 mm Hg; diastolic, <80 mm Hg), elevated (systolic, 120–129 mm Hg; diastolic, <80 mm Hg), stage 1 HTN (systolic 130–139 mm Hg; diastolic, 80–89 mm Hg), and stage 2 HTN (systolic, ≥140 mm Hg; diastolic, ≥90 mm Hg). Four conditions were established on the premise that participants classified into each category stayed in the same category during the 10-year observation period. First, only participants classified into the same BP category in 2008 and at previous health examinations (2005, 2006, or 2007) were enrolled in this study. Second, only participants remained in the same BP category at the additional health examination for the previous 3 years, including the year of stroke occurrence, were included in this study [e.g., participants who had a stroke in 2011 should have had additional health examination data after January (JAN) 2009; participants who had a stroke in 2017 should have had additional health examination data after January 2015]. Third, participants who had a stroke after January 2014 or remained stroke-free during the observation period were included in this study only if they underwent an additional health examination between 2009 and 2013, and their BP category remained unchanged and the same. Fourth, participants who had no stroke during the 10-year observation period had to have undergone additional health examinations at least once in both 2009–2013 and 2014–2018, and all had to have been classified into the same BP group as that of the first examination. Therefore, only participants classified in the same category three to four times during the health examinations were included in this study. Finally, only patients who did not take BP medication before study enrollment until stroke occurred were included to assess the natural association between BP categories and stroke risk.

### Primary outcomes

Primary outcomes were HS, including ICH and SAH, and secondary outcomes were analyzed by dividing the participants into those with ICH and those with SAH. The ICH (ICD-10 code I61) and SAH (ICD-10 code I60) groups included only patients who were hospitalized for more than 5 days or died within 5 days with an ICD-10 code. Since the risk for stroke differs by sex (12–16), each variable was analyzed by classifying participants into men and women.

### Statistical analysis

Participants were followed up from 1 January 2009, until the date of an HS or 31 December 2018. The multivariable-adjusted hazard ratio (HR) and 95% confidence intervals (CIs) for ICH, SAH, and HS were determined by Cox proportional hazard regression analysis according to BP after adjustments for all covariates. The variables included sex, age (categorical, <40, 40–49, 50–59, and 60–64 years), BP (categorical, low normal, high normal, elevated, stage 1 HTN, and stage 2 HTN), body mass index (BMI) (categorical, <18.5, 18.5–22.9, 23.0–24.9, 25.0–29.5, and ≥30), fasting serum glucose (FSG) (categorical, <100, 100–125.9, and ≥126 mg/dl), total cholesterol (categorical, <200, 200–239.9, and ≥240 mg/dl), heart disease (categorical, yes or no), family history [e.g., HTN, stroke, and diabetes mellitus (DM)] (categorical, yes or no), alcohol consumption (categorical, 0, <1, 1–2, 3–4, and ≥5 times per week), current smoking (categorical, yes or no), and physical activity (categorical, 0, 1–2, 3–4, 5–6, and 7 times per week). Cox proportional hazard regression analysis was performed for all covariates to identify risk factors for HS. Multivariable analysis was performed for variables with a *p*-value <0.1 in univariable analysis and was fitted using the backward selection method to eliminate interactions between variables. The Grambsch–Therneau method based on the Schoenfeld residual was tested, and the proportional hazard assumptions for all selected variables were satisfied with a *p*-value of >0.05. Missing data were excluded. Data manipulation and extraction were performed using SAS software, version 9.4 (SAS Institute Inc., Cary, NC, USA).

## Results

### Baseline characteristics

**Figure 1:**
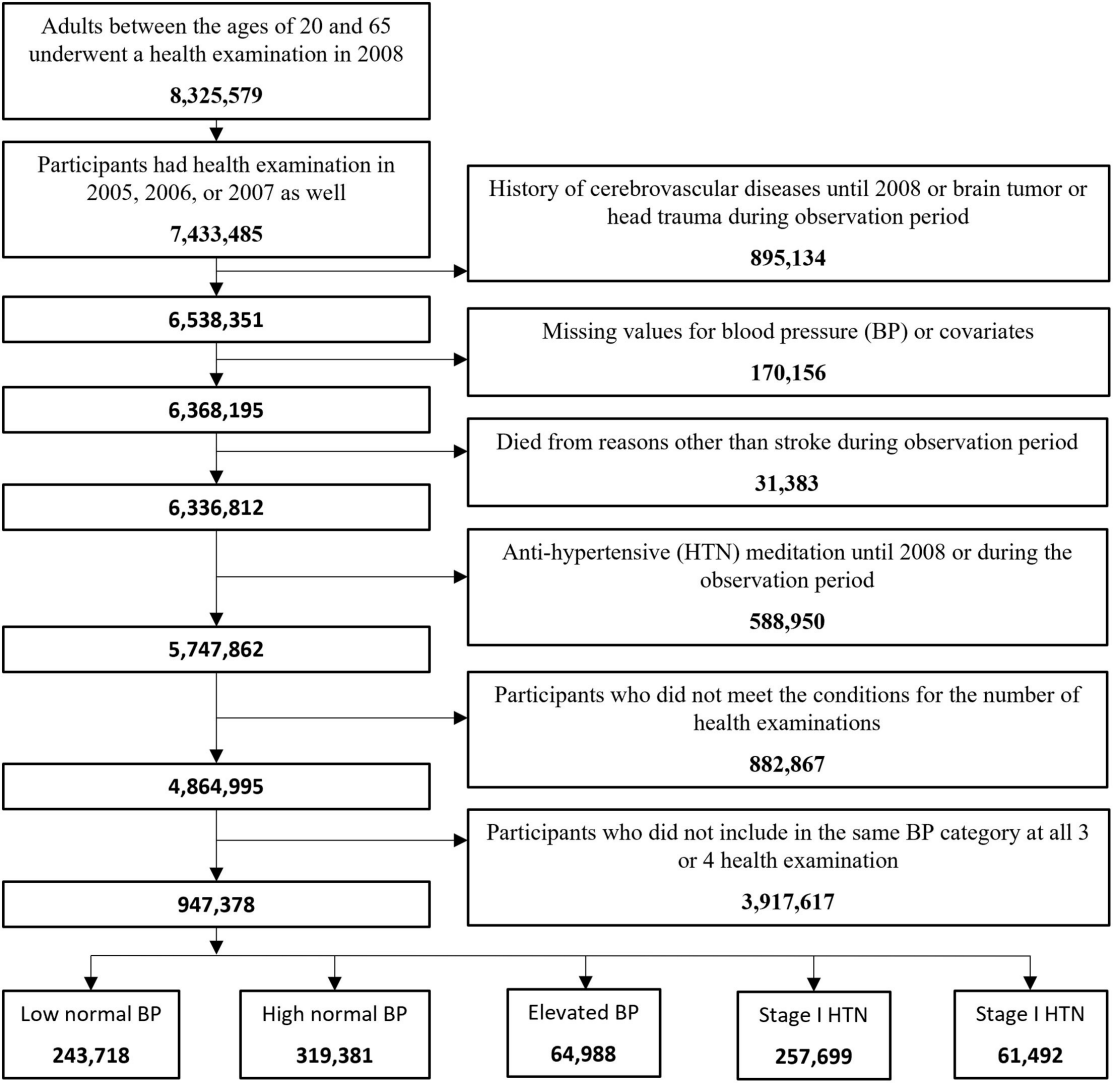
Flowchart of patient registration.

In 2008, 8,325,579 adults between the ages of 20 and 65 underwent a health examination. Among them, 7,433,485 participants had health examinations in 2005, 2006, or 2007. Excluding those who had a history of stroke until 2008 or who had a brain tumor or head trauma during the observation period out of 7,433,485 participants, 6,538,351 remained. Of those, 170,156 participants (2.6%) with missing values for BP or missing covariates were excluded. A total of 31,383 participants (0.49%) who died from reasons other than CVD during the observation period were also excluded. Among 6,336,812 participants, 588,950 (9.3%) who had taken antihypertensive medications until 2008 or took antihypertensive medications during the observation period were also excluded. Among 4,864,995 participants who met the conditions for the number of health examinations during the observation period, the number of participants included in the same BP category at all three or four health examinations was 947,378. These were finally enrolled in this study and classified into the low normal group, high normal group, elevated group, stage 1 HTN group, and stage 2 HTN group with 243,718, 319,381, 64,988, 257,699, and 61,492 participants, respectively (Figure 1).

Among 528,178 men and 419,200 women, HS occurred in 560 (0.11%) and 353 (0.084%) patients, respectively. Among men, age, BP, FSG, and alcohol consumption in the HS group showed significantly higher trends than in the non-HS group, and in the case of women, age, BP, and BMI in the HS group showed significantly higher trends than in the non-HS group. The proportion of current smokers was significantly higher in the HS group for both men and women. Table 1 depicts the descriptive characteristics of the study population.

**Table 1:** Descriptive characteristics of the study population.

	**Total**	**Men**			**Women**		
		**Non-hSTK group (%) (*****n** =* **527,618)**	**hSTK group (%) (*****n*** = **560)**	**p-value**	**Non-hSTK group (%) (*****n*** = **418,847)**	**hSTK group (%) (*****n*** = **353)**	**p-value**
Age (mean ± SD, years)^*^		38.01 ± 9.46	42.97 ± 10.12	<0.0001	36.57 ± 10.67	38.01 ± 9.46	<0.0001
<40	562,542	315,527 (59.74)	226 (40.36)		246,690 (58.9)	99 (28.05)	
40–49	258,117	140,371 (26.58)	180 (32.14)		117,404 (28.03)	162 (45.89)	
50–59	105,885	60,095 (11.38)	116 (20.71)		45,606 (10.89)	68 (19.26)	
60–64	20,834	11,625 (2.2)	38 (6.79)		9,147 (2.18)	24 (6.8)	
Blood pressure (SBP or/and DBP, mmHg)				<0.0001			<0.0001
<110 and <80	243,718 (25.73)	58,579 (11.10)	55 (9.82)		184,981 (44.16)	103 (29.18)	
110–119 and <80	319,381 (33.72)	172,175 (32.63)	115 (20.54)		147,085 (35.12)	106 (30.03)	
120–129 and <80	64,988 (6.86)	44,176 (8.36)	44 (7.86)		20,749 (4.95)	19 (5.38)	
130–139 or 80–89	257,699 (27.10)	203,348 (38.5)	220 (39.29)		54,036 (12.9)	95 (26.91)	
≥140 or ≥90	61,492 (6.49)	493,40 (9.34)	126 (22.5)		11,996 (2.86)	30 (8.5)	
Body mass index				0.0511			<0.0001
<18.5	55,836 (5.89)	12,726 (2.41)	18 (3.21)		43,075 (10.28)	17 (4.82)	
18.5–22.9	516,656 (54.54)	232,821 (44.13)	260 (46.43)		283,357 (67.65)	218 (61.76)	
23.0–24.9	168,064 (17.74)	119,621 (22.67)	105 (18.75)		48,276 (11.53)	62 (17.56)	
25.0–29.5	191,468 (20.21)	150,728 (28.57)	158 (28.21)		40,527 (9.68)	55 (15.58)	
≥30	15,354 (1.62)	11,722 (2.22)	19 (3.39)		3,612 (0.86)	1 (0.28)	
Fasting serum glucose (mg/dL)				0.0132			0.0725
<100.0	851,882 (89.92)	452,552 (85.77)	469 (83.75)		398,532 (95.15)	329 (93.2)	
100.0–125.9	81,041 (8.55)	63,114 (11.96)	68 (12.14)		17,840 (4.26)	19 (5.38)	
≥126.0	14,455 (1.53)	11,952 (2.27)	23 (4.11)		2,475 (0.59)	5 (1.42)	
Total cholesterol (mg/dL)				0.6540			0.4133
<200.0	731,733 (77.24)	384,097 (72.8)	399 (71.25)		346,954 (82.84)	283 (80.17)	
200.0-239.9	173,899 (18.36)	114,955 (21.79)	131 (23.39)		58,756 (14.03)	57 (16.15)	
≥240.0	41,746 (4.41)	28,566 (5.41)	30 (5.36)		13,137 (3.14)	13 (3.68)	
Heart disease				0.3760			0.2973
No	944,001 (99.64)	525,398 (99.58)	559 (99.82)		417,693 (99.43)	351 (99.43)	
Yes	3,377 (0.36)	2,220 (0.42)	1 (0.18)		1,154 (0.28)	2 (0.57)	
**Family history**							
Hypertension				0.9062			0.1173
No	863,723 (91.17)	486,864 (92.28)	516 (92.14)		376,035 (89.78)	308 (87.25)	
Yes	83,655 (8.83)	40,754 (7.72)	44 (7.86)		42,812 (10.21)	45 (12.75)	
Stroke				0.1334			0.3259
No	908,673 (95.91)	505,991 (95.9)	530 (94.64)		401,817 (95.93)	335 (94.9)	
Yes	38,705 (4.09)	21,627 (4.1)	30 (5.36)		17,030 (4.07)	18 (5.1)	
Diabetes mellitus				0.2538			0.5959
No	874,351 (92.29)	489,974 (92.87)	527 (94.11)		383,524 (91.57)	326 (92.35)	
Yes	73,027 (7.71)	37,644 (7.13)	33 (5.89)		35,323 (8.43)	27 (7.65)	
Alcohol consumption (times/week)				<0.0001			0.0896
0	451,605 (47.67)	175,564 (33.27)	168 (30.00)		275,626 (65.81)	247 (69.97)	
<1	231,719 (24.46)	138,166 (26.19)	110 (19.64)		93,372 (22.29)	71 (20.11)	
1–2	207,531 (21.91)	163,600 (31.01)	194 (34.64)		43,707 (10.44)	30 (8.5)	
3–4	46,415 (4.90)	41,397 (7.85)	63 (11.25)		4,953 (1.18)	2 (0.57)	
≥5	10,108 (1.07)	8,891 (1.68)	25 (4.46)		1,189 (0.28)	3 (0.85)	
Current smoker				0.0009			0.0215
No	709,876 (74.93)	299,536 (56.77)	279 (49.82)		409,722 (97.82)	339 (96.03)	
Yes	237,502 (25.07)	228,082 (43.23)	281 (50.18)		9,125 (2.18)	14 (3.97)	
Physical activity (times/week)				0.5238			0.0589
0	501,814 (52.97)	229,395 (43.48)	254 (45.36)		271,951 (64.93)	214 (60.62)	
1–2	293,452 (30.98)	200,536 (38.01)	212 (37.86)		92,626 (22.11)	78 (22.1)	
3–4	106,141 (11.20)	68,982 (13.07)	64 (11.43)		37,055 (8.84)	40 (11.33)	
5–6	20,982 (2.21)	12,875 (2.44)	10 (1.79)		8,084 (1.93)	13 (3.68)	
7	24,989 (2.64)	15,830 (3.00)	20 (3.57)		9,131 (2.18)	8 (2.27)	

DBP, diastolic blood pressure; hSTK, hemorrhagic stroke; SBP, systolic blood pressure.

^*^Start age of observation.

### Risk of intracerebral hemorrhage and subarachnoid hemorrhage

The incidence and risk of ICH and SAH according to BP categories are shown in Table 2. Compared with men with low normal BP, those with elevated stage 2 HTN [incidence, 9.1 vs. 4.1 per 100,000 person-years; adjusted HR (AHR), 2.002 (95% CI 1.203–3.332)] had a significantly elevated risk of ICH. Similarly, compared with women with low normal BP, those with stage 1 HTN [incidence, 5.4 vs. 2.7 per 100,000 person-years; AHR, 2.021 (95% CI, 1.251–3.263)] and stage 2 HTN [incidence, 7.5 vs. 2.7 per 100,000 person-years; AHR, 2.755 (95% CI, 1.270–5.977)] had a significantly elevated risk of ICH.

**Table 2:** Risk of intracerebral hemorrhage and subarachnoid hemorrhage according to categories of blood pressure.

	**Systolic/diastolic blood pressure measures, mm Hg**				
	**Normal**		**Elevated**	**HTN, Stage 1**	**HTN, Stage 2**
	**SBP**<**110 and DBP**<**80**	**SBP 110–119 and DBP**<**80**	**SBP 120–129 and DBP**<**80**	**SBP 130–139 or DBP 80–89**	**SBP** ≥**140 or DBP** ≥**90**
**Intracerebral hemorrhage: men**					
Events	29	50	17	83	45
Person-years	585,964	1,722,041	441,848	2,033,979	493,641
Incidence (events/100,000 person-years)	4.9	2.9	3.8	4.1	9.1
Adjusted HR (95% CI)^*^	1 (ref)	0.681 (0.429–1.080)	0.949 (0.518–1.740)	1.004 (0.647–1.558)	2.002 (1.203–3.332)
**Intracerebral hemorrhage: women**					
Events	50	36	7	29	9
Person-years	1,850,097	1,471,052	207,529	540,539	120,000
Incidence (events/100,000 person-years)	2.7	2.4	3.4	5.4	7.5
Adjusted HR (95% CI)^*^	1 (ref)	0.913 (0.593–1.404)	1.242 (0.559–2.761)	2.021 (1.251–3.263)	2.755 (1.270–5.977)
**Subarachnoid hemorrhage: men**					
Events	26	65	27	138	83
Person-years	585,953	1,722,100	441,910	2,034,328	493,786
Incidence (events/100,000 person-years)	4.4	3.8	6.1	6.8	16.8
Adjusted HR (95% CI)^*^	1 (ref)	0.886 (0.561–1.399)	1.478 (0.859–2.544)	1.637 (1.066–2.514)	4.217 (2.648–6.715)
**Subarachnoid hemorrhage: women**					
Events	54	71	12	69	21
Person-years	1,850,131	1,471,259	207,553	540,738	120,071
Incidence (events/100,000 person-years)	2.9	4.8	5.8	12.8	17.5
Adjusted HR (95% CI)^*^	1 (ref)	1.499 (0.956–2.262)	1.775 (0.944–3.34)	3.902 (2.688–5.666)	5.166 (3.000–8.896)

CI, confidence interval; DBP, diastolic blood pressure; HR, harzard ratio; HTN, hypertension; SBP, systolic blood pressure.

^*^Hazard ratio calculated by Cox proportional hazards regression analysis after adjustments for age, body mass index, fasting serum glucose, total cholesterol, heart disease, family history, smoking, physical activity, alcohol consumption.

Regarding SAH, compared with men with low normal BP, those with stage 1 HTN [incidence, 6.8 vs. 4.4 per 100,000 person-years; AHR, 1.637 (95% CI, 1.066–2.514)] and stage 2 HTN [incidence, 16.8 vs. 4.4 per 100,000 person-years; AHR, 4.217 (95% CI, 2.648–6.715)] had a significantly higher risk. Similarly, compared with women with low normal BP, those with stage 1 HTN [incidence, 12.8 vs. 2.9 per 100,000 person-years; AHR, 3.902 (95% CI, 2.688–5.666)] and stage 2 HTN [incidence, 17.5 vs. 2.9 per 100,000 person-years; AHR, 5.166 (95% CI, 3.000–8.896)] had a significantly higher risk of SAH.

### Risk of hemorrhagic stroke

The incidence and risk of HS, including ICH and SAH, according to BP categories are shown in Table 3. Compared with men with low normal BP, men with stage 2 HTN [incidence, 25.5 vs. 9.4 per 100,000 person-years; AHR, 3.034 (95% CI, 2.161–4.260)] had a significantly higher risk of HS. Similarly, women with stage 1 HTN [incidence, 17.6 vs. 5.6 per 100,000 person-years; AHR, 2.976 (95% CI, 2.222–3.986)] and stage 2 HTN [incidence, 25.0 vs. 5.6 per 100,000 person-years; AHR, 4.132 (95% CI, 2.659–6.421)] had a significantly higher risk of developing HS.

**Table 3:** Risk of hemorrhagic stroke according to categories of blood pressure.

	**Systolic/diastolic blood pressure measures, mm Hg**				
	**Normal**		**Elevated**	**HTN, Stage 1**	**HTN, Stage 2**
	**SBP**<**110 and DBP**<**80**	**SBP 110–119 and DBP**<**80**	**SBP 120–129 and DBP**<**80**	**SBP 130–139 or DBP 80–89**	**SBP** ≥**140 or DBP** ≥**90**
**Hemorrhagic stroke: men**					
Events	55	115	44	220	126
Person-years	586,127	1,722,391	441,998	2,034,820	494,020
Incidence (events/100,000 person-years)	9.4	6.7	10.0	10.8	25.5
Adjusted HR (95% CI)^*^	1 (ref)	0.778 (0.563–1.075)	1.209 (0.810–1.804)	1.310 (0.966–1.776)	3.034 (2.161–4.260)
**Hemorrhagic stroke: women**					
Events	103	106	19	95	30
Person-years	1,850,408	1,471,479	207,592	540,892	120,111
Incidence (events/100,000 person-years)	5.6	7.2	9.2	17.6	25.0
Adjusted HR (95% CI)^*^	1 (ref)	1.270 (0.967–1.669)	1.538 (0.938–2.521)	2.976 (2.222–3.986)	4.132 (2.659–6.421)

CI, confidence interval; DBP, diastolic blood pressure; HR, harzard ratio; HTN, hypertension; SBP, systolic blood pressure.

^*^Hazard ratio calculated by Cox proportional hazards regression analysis after adjustments for age, body mass index, fasting serum glucose, total cholesterol, heart disease, family history, smoking, physical activity, alcohol consumption.

### Evaluation of risk factors for hemorrhagic stroke

Age, BP, BMI, FSG, total cholesterol, history of heart disease, family history (HTN, stroke, and DM), alcohol consumption, smoking status, and exercise habits were included in the initial model. To evaluate risk factors for HS in men, after univariable analysis, four variables (total cholesterol, heart disease, family history, and exercise habits) were removed (Table 4). Advanced age [ <40, Ref; 40–49, HR 1.754 (95% CI 1.436–2.144); 50–59, HR 2.508 (95% CI 1.983–3.172); 60–64, HR 4.087 (95% CI 2.857–5.847)], stage 2 HTN [HR 2.699 (95% CI 1.926–3.783)], heavy alcohol consumption [≥5 times/week, HR 1.794 (95% CI 1.166–2.761)], and current smoking [HR 1.388 (95% CI 1.163–1.657)] were found to be independent risk factors for HS among men. Normal BMI was identified as a protective factor [23.0–24.9, HR 0.506 (95% CI 0.304–0.839)] for HS among men (Table 4).

**Table 4:** Univariable and multivariable analysis to identify predictive factors for hemorrhagic stroke in males.

	**Univariable analysis**			**Multivariable analysis**		
	**HR**	**95% CI**	**p-value**	**HR**	**95% CI**	**p-value**
**Age** ^*^						
<40	Ref			Ref		
40–49	1.790	1.471–2.177	<0.0001	1.754	1.436–2.144	<0.0001
50–59	2.693	2.153–3.369	<0.0001	2.508	1.983–3.172	<0.0001
60–64	4.559	3.233–6.428	<0.0001	4.087	2.857–5.847	<0.0001
**Blood pressure (SBP or/and DBP, mmHg)**						
<110 and <80	Ref			Ref		
110–119 and <80	0.712	0.516–0.981	0.0380	0.83	0.600–1.148	0.2599
120–129 and <80	1.061	0.714–1.577	0.7702	1.263	0.846–1.886	0.2535
130–139 or 80–89	1.152	0.857–1.548	0.3474	1.326	0.978–1.797	0.0693
≥140 or ≥90	2.72	1.981–3.733	<0.0001	2.699	1.926–3.783	<0.0001
**Body mass index**						
<18.5	Ref			Ref		
18.5–22.9	0.790	0.490–1.273	0.3328	0.734	0.454–1.186	0.2064
23.0–24.9	0.621	0.377–1.024	0.0617	0.506	0.304–0.839	0.0084
25.0–29.5	0.741	0.455–1.207	0.2291	0.545	0.331–1.198	0.1072
≥30	1.146	0.602–2.184	0.6784	0.794	0.410–1.536	0.4929
**Fasting serum glucose (mg/dL)**						
<100.0	Ref			Ref		
100.0–125.9	1.04	0.806–1.341	0.7645	0.721	0.555–1.137	0.0944
≥126.0	1.856	1.222–2.821	0.0038	0.993	0.646–1.524	0.9728
**Total cholesterol (mg/dL)**						
<200.0	Ref					
200.0–239.9	1.097	0.901–1.336	0.3579			
≥240.0	1.011	0.698–1.465	0.9540			
Heart disease	0.424	0.060–3.012	0.3909			
**Family history**						
Hypertension	1.019	0.749–1.386	0.9054			
Stroke	1.325	0.917–1.914	0.1336			
Diabetes mellitus	0.815	0.579–1.159	0.2549			
**Alcohol consumption (times/week)**						
0	Ref			Ref		
<1	0.832	0.654–1.058	0.1338	0.86	0.673–1.099	0.2283
1–2	1.239	1.008–1.523	0.0420	1.151	0.926–1.430	0.2065
3–4	1.590	1.190–2.124	0.0017	1.255	0.928–1.696	0.1405
≥5	2.935	1.928–4.468	<0.0001	1.794	1.166–2.761	0.0079
Current smoker	1.322	1.121–1.561	0.0009	1.388	1.163–1.657	0.0003
**Physical activity (times/week)**						
0	Ref					
1–2	0.955	0.796–1.146	0.6188			
3–4	0.838	0.637–1.102	0.2063			
5–6	0.702	0.373–1.320	0.2725			
7	1.141	0.724–1.799	0.5698			

CI, confidence interval; DBP, diastolic blood pressure; HR, harzard ratio; HTN, hypertension; SBP, systolic blood pressure.

^*^Start age of observation.

Table 5 depicts the risk factors for HS among women. Univariable analysis eliminated three variables (total cholesterol, heart disease, and family history). After multivariable analysis, advanced age [ <40, Ref; 40–49, HR 3.103 (95% CI 2.381–4.043); 50–59, HR 2.964 (95% CI 2.115–4.154); 60–64, HR 4.733 (95% CI 2.934–7.637)], stage 1 HTN [low normal, Ref; HR 2.491 (95% CI 1.858–3.339)], stage 2 HTN [low normal, Ref; HR 2.875 (95% CI 1.851–4.466)], and current smoking [HR 2.633 (95% CI 1.538–4.506)] were demonstrated to be significant risk factors for HS among women. There was a significantly higher risk of SAH among women than among men [HR 1.590 (95% CI 1.255–2.015)] despite no significant difference for HS (*p* = 0.1106) and ICH (*p* = 0.1445).

**Table 5:** Univariable and multivariable analysis to identify predictive factors for hemorrhagic stroke in females.

	**Univariable analysis**			**Multivariable analysis**		
	**HR**	**95% CI**	**p-value**	**HR**	**95% CI**	**p-value**
**Age** ^*^						
<40	Ref			Ref		
40–49	3.437	2.676–4.413	<0.0001	3.103	2.381–4.043	<0.0001
50–59	3.713	2.727–5.056	<0.0001	2.964	2.115–4.154	<0.0001
60–64	6.530	4.181–10.199	<0.0001	4.733	2.934–7.637	<0.0001
**Blood pressure (SBP or/and DBP, mmHg)**						
<110 and <80	Ref			Ref		
110–119 and <80	1.294	0.987–1.697	0.0624	1.275	0.970–1.674	0.0812
120–129 and <80	1.644	1.008–2.682	0.0464	1.316	0.802–2.159	0.2777
130–139 or 80–89	3.155	2.388–4.170	<0.0001	2.491	1.858–3.339	<0.0001
≥140 or ≥90	4.487	2.988–6.739	<0.0001	2.875	1.851–4.466	<0.0001
**Body mass index**						
<18.5	Ref			Ref		
18.5–22.9	1.949	1.190–3.192	0.0081	1.240	0.749–2.053	0.4021
23.0–24.9	3.253	1.902–5.562	<0.0001	1.305	0.744–2.288	0.3537
25.0–29.5	3.437	1.995–5.921	<0.0001	1.128	0.633–2.009	0.6838
≥30	0.703	0.094–5.272	0.7317	0.212	0.028–1.611	0.1338
**Fasting serum glucose (mg/dL)**						
<100.0	Ref			Ref		
100.0–125.9	1.290	0.812–2.048	0.2806	0.763	0.476–1.223	0.2616
≥126.0	2.446	1.011–5.916	0.0471	1.180	0.483–2.886	0.7162
**Total cholesterol (mg/dL)**						
<200.0	Ref					
200.0–239.9	1.189	0.895–1.581	0.2325			
≥240.0	1.213	0.696–2.115	0.4959			
Heart disease	2.062	0.514–8.272	0.3070			
**Family history**						
Hypertension	1.283	0.939–1.755	0.1179			
Stroke	1.268	0.789–2.037	0.3265			
Diabetes mellitus	0.899	0.607–1.332	0.5962			
**Alcohol consumption (times/week)**						
0	Ref			Ref		
<1	0.849	0.652–1.105	0.2227	0.962	0.804–1.152	0.6746
1–2	0.766	0.524–1.119	0.1681	1.081	0.858–1.361	0.5102
3–4	0.451	0.112–1.812	0.2617	0.790	0.391–1.596	0.5116
≥5	2.813	0.902–8.780	0.0748	1.581	0.706–3.540	0.2657
Current smoker	1.854	1.087–3.164	0.0236	2.633	1.538–4.506	0.0004
**Physical activity (times/week)**						
0	Ref			Ref		
1–2	1.070	0.826–1.387	0.6083	0.920	0.708–1.194	0.5296
3–4	1.372	0.979–1.922	0.0666	1.033	0.734–1.453	0.8520
5–6	2.043	1.167–3.576	0.0124	1.435	0.818–2.520	0.2080
7	1.113	0.550–2.255	0.7655	0.662	0.326–1.347	0.2552

CI, confidence interval; DBP, diastolic blood pressure; HR, harzard ratio; HTN, hypertension; SBP, systolic blood pressure.

^*^Start age of observation.

## Discussion

### Previous studies on stroke with intensive blood pressure control

The association between intensive BP control and stroke incidence has been studied in several randomized controlled trials (RCTs). In 2010, the Action to Control Cardiovascular Risk in Diabetes (ACCORD) study, which included 4,733 type 2 DM patients with an intensive BP control group (SBP <120 mm Hg) and a standard BP control group, showed that a significant result in the HR of stroke in the intensive BP control group was lower than that of the standard BP control group [HR 0.59 (95% CI 0.39–0.89)] (17). In 2013, the Secondary Prevention of Small Subcortical Strokes (SPS3) trial, conducted with 3,020 patients with lacunar stroke within 6 months to prevent secondary stroke, demonstrated that only ICH showed a significant HR reduction in the lower BP group under 130 mm Hg [HR 0.37 (95% CI 0.15–0.95)] (10). In 2015, the Systolic Blood Pressure Intervention Trial (SPRINT) that was performed with 9,361 non-diabetic patients without a prior stroke history showed no significant benefit of intensive BP in the prevention of primary stroke [HR 0.89 (95% CI 0.63–1.25)] (18). The next year, the SPRINT research group published two other subgroup analyses, and these studies announced that intensive BP control did not significantly reduce all types of stroke or overall stroke even in the elderly population aged over 75 years (19, 20). In 2018, the Recurrent Stroke Prevention Clinical Outcome (RESPECT) study, conducted with 1,263 patients with a stroke history within 3 years of stroke to determine the effectiveness of intensive BP control in the prevention of secondary stroke, demonstrated that only ICH showed statistical significance [HR 0.09 (95% CI 0.01–0.70)] (3). Finally, in 2021, the Strategy of Blood Pressure Intervention in the Elderly Hypertensive Patients (STEP) trial reported the stroke prevention effect of intensive BP control (SBP <120 mm Hg) compared with standard BP control for 8,511 Chinese individuals aged 60–80 years (21). The results showed that intensive BP control had a significant preventive effect on stroke compared to standard BP control [HR 0.67 (95% CI 0.47–0.97)].

Overall, the STEP trial (18) was the only study that showed that intensive BP control had a preventive effect on primary stroke in the population without a history of stroke, but this study had limitations in that it included only elderly individuals between the ages of 60 and 80 years. Although the stroke incidence in the intensive BP control group in SPRINT (18) showed a tendency to be low, the reason for not obtaining a significant result for stroke was that the incidence of stroke was lower than that of cardiovascular events, so there is a possibility that the number of subjects and the observation period were insufficient to obtain a significant result. Therefore, this study was conducted to determine the risk of primary stroke, especially HS, according to BP during a long observation period of 10 years in the population aged between 20 and 65 years without a history of stroke.

### Risk of ICH and SAH according to sex and blood pressure

This study demonstrated that the AHR for ICH increased significantly for women with stage 1 HTN although that of ICH increased significantly for men with stage 2 HTN. This result seems to be contradictory to previous studies that showed a higher risk of ICH among men than among women (22). The reason for this sex difference in this study is that since the incidence of ICH among men with low normal BP, which was the reference BP in this study, was higher than that among women, men with stage 1 HTN did not show a statistically significant difference in the AHR for ICH although men generally have a higher incidence of ICH than women. Therefore, it should not be misunderstood that men have a preventive effect on ICH compared to women. As shown in Table 1, the proportion of DM, hypercholesterolemia, smoking, and alcohol consumption reported as risk factors for ICH tended to be higher among men than among women, and these factors were identified as the cause of the higher incidence of ICH among men than among women in the same BP group (22). Nevertheless, there was no significant difference in risk between men and women regarding the incidence of ICH in this study.

On the other hand, the AHR for the incidence of SAH was significantly increased for both men and women with stage 1 HTN. Among women with stage 1 HTN, the incidence of SAH was almost twice as high as that among men. The reason can be considered based on the study by Kim et al., who analyzed the epidemiology of unruptured intracranial aneurysm (UIA) using the same NHIS database (23). According to that study, the HR of women for the incidence of UIA was reported to be 1.56 (95% CI 1.34–1.81), and in this study, the AHR of women for SAH was 1.590 (95% CI 1.255–2.015). Therefore, it can be assumed that the high incidence of UIA among women is one of the main reasons for the significantly higher incidence of SAH among women with stage 1 HTN. This is similar to the HR of SAH for women reported in the previous literature because estrogen deficiency affects both UIA formation and SAH after menopause (24, 25).

### Risk of hemorrhagic stroke according to sex and blood pressure and optimal blood pressure target for preventing stroke

For overall HS, men with stage 2 HTN showed a significant increase in incidence, and women with stage 1 HTN showed a significant increase in incidence as well. Therefore, for the primary prevention of HS, men need to manage a SBP of <140 mm Hg and a DBP of <90 mm Hg, and women need to manage a SBP of <130 mm Hg and a DBP of <80 mm Hg. However, for SAH, men with stage 1 HTN also had a statistically significant increase in the AHR. Accordingly, these results indicate that it is reasonable to start BP management from stage 1 HTN for both men and women to prevent ICH or SAH, the leading causes of HS. According to the risk factors for HS demonstrated in this study, more attention should be given to BP management at advanced ages since the risk of HS increases significantly with age for both men and women. For women, BP management is more important at advanced ages because an increase in HR with age is greater than that for men due to the effects of estrogen deficiency after menopause (25). On the other hand, for men, weight management is important since normal BMI has a protective effect, and men are recommended to stop smoking and drinking excessively. In several previous studies, advanced age, hypertension, obesity, smoking, and excessive alcohol consumption were significant risk factors for HS, which is consistent with our results (25–33). However, only smoking was a significant risk factor for HS among women in this study, which is assumed to be because the statistical power of these factors was not sufficient because women have a much lower rate of obesity and alcohol consumption than men in Korea.

### Limitations

The several limitations of this study are that it is an observational study using NHIS data rather than an RCT. Second, despite the premise that BP control was consistent during the 10-year observation period, based on the study design in which only subjects classified into the same blood pressure group were enrolled in the study for a total of 3–4 checkups, it is also possible that intraday fluctuations in BP or white coat HTN may have influenced the results. Third, since only those who had not taken HTN medications for 10 years were included in this study, there may be a selection bias in that these participants are relatively less strict about their healthcare. Finally, it is essential to recognize that the study population solely represents individuals from a single country. Consequently, the generalizability of the findings to other racial or ethnic groups should be interpreted with caution, considering potential geographic and racial disparities in stroke incidence and risk factors.

Despite these limitations, the study has notable strengths, primarily its robust statistical power, which involved nearly 1 million patients over a 10-year observation period. This extensive dataset has provided valuable insights and yielded statistically significant results for certain aspects of hemorrhagic stroke that were not previously demonstrated in randomized controlled trials (RCTs). As with any research, it is crucial to have a comprehensive understanding of both the strengths and limitations to interpret the implications and practical applications of the findings effectively.

## Conclusion

This study demonstrated the risk of HS and its subtypes according to BP classification based on 2017 ACC/AHA guidelines by sex in the healthy young adult population. In conclusion, to prevent HS, both men and women between the ages of 20 and 65 years should manage their BP starting from stage 1 HTN, and stricter BP management should be required at advanced ages. In addition, smoking should be avoided, especially for men, who should also avoid excessive drinking and maintain a normal weight.

## Data availability statement

Due to the stringent privacy regulations enforced by the NHIS, the raw data cannot be made publicly accessible. However, processed data used in the analysis phase is exempt from these restrictions and can be found in the Supplementary files associated with this paper.

## Ethics statement

The studies involving humans were approved by the Seoul National University Bundang Hospital Institutional Review Board (IRB number: X-1910-572-903). The studies were conducted in accordance with the local legislation and institutional requirements. Written informed consent for participation was not required from the participants or the participants' legal guardians/next of kin because due to the retrospective nature of this study.

## Author contributions

HSS: Visualization, Writing—original draft. J-MP: Writing—original draft, Writing—review and editing. YJL: Data curation. Y-DK: Data curation. TK: Methodology. SPB: Formal analysis. JSB: Formal analysis, Methodology. O-KK: Supervision, Writing—review and editing. CWO: Supervision, Writing—review and editing. SUL: Conceptualization, Funding acquisition, Methodology, Project administration, Supervision, Writing—review and editing.
