# Supplementary material for: Optimal target blood pressure for the primary prevention of hemorrhagic stroke: a nationwide observational study
Source: Front Neurol. 2023 Oct 9;14:1268542. doi: 10.3389/fneur.2023.1268542 (PMC10593468; doi:10.3389/fneur.2023.1268542)
Supplement: Supplementary file 1 [file Data_Sheet_1.PDF]

The TTEST Procedure

Variable: AGE

OUTC	Method	N	Mean	Std Dev	Std Err	Minimum	Maximum
0		527618	38.098	9.4619	0.013	16	64
1		560	42.9714	10.1173	0.4275	20	64
Diff (1-2)	Pooled		-4.8735	9.4627	0.4001		
Diff (1-2)	Satterthwaite		-4.8735		0.4277		

OUTC	Method	Mean	95% CL Mean		Std Dev	95% CL Std Dev	
0		38.098	38.0724	38.1235	9.4619	9.4439	9.48
1		42.9714	42.1317	43.8112	10.1173	9.5575	10.7474
Diff (1-2)	Pooled	-4.8735	-5.6576	-4.0893	9.4627	9.4447	9.4807
Diff (1-2)	Satterthwaite	-4.8735	-5.7136	-4.0333			

Method	Variances	DF	t Value	Pr > |t|
Pooled	Equal	528176	-12.18	<.0001
Satterthwaite	Unequal	560.04	-11.39	<.0001

Equality of Variances

Method	Num DF	Den DF	F Value	Pr > F
Folded F	559	527617	1.14	0.021

FREQ Procedure

Table OUTC * AGE_1

OUTC		AGE_1 0	AGE_1 1	AGE_1 2	AGE_1 3	Total
0	Frequency	315527	140371	60095	11625	527618
	Expected value	315418	140402	60147	11651	
	Percentage	59.74	26.58	11.38	2.2	99.89
	Row Percentage	59.8	26.6	11.39	2.2	
	Coloum Percentage	99.93	99.87	99.81	99.67	
1	Frequency	226	180	116	38	560
	Expected value	334.78	149.02	63.839	12.366	
	Percentage	0.04	0.03	0.02	0.01	0.11
	Row Percentage	40.36	32.14	20.71	6.79	
	Coloum Percentage	0.07	0.13	0.19	0.33	
Total	Frequency	315753	140551	60211	11663	528178
	Percentage	59.78	26.61	11.4	2.21	100

OUTC * AGE_1 Statistics for the table

Statistics	Degree of freedom	Value	Prob
Chi square	3	137.6916	<.0001
Likelihood ratio Chi square	3	114.4202	<.0001
Mantel-Haenszel Chi square	1	132.652	<.0001
pi coefficient		0.0161	
Contingency coefficient		0.0161	
Kramer's V		0.0161	

Sample size = 528178

FREQ Procedure

Table OUTC * SEX_TYPE_1

		SEX_TYPE_1	
		1	Total
OUTC			
0	Frequency	527618	527618
	Expected value	527618	
	Percentage	99.89	99.89
	Row Percentage	100	
	Coloum Percentage	99.89	
1	Frequency	560	560
	Expected value	560	
	Percentage	0.11	0.11
	Row Percentage	100	
	Coloum Percentage	0.11	
Total	Frequency	528178	528178
	Percentage	100	100

FREQ Procedure

Table OUTC * BP_1

OUTC		BP_1: 0	1	2	3	4	Total
0	Frequency	58579	172175	44176	203348	49340	527618
	Expected value	58572	172107	44173	203352	49414	
	Percentage	11.09	32.6	8.36	38.5	9.34	99.89
	Row Percentage	11.1	32.63	8.37	38.54	9.35	
	Coloum Percentage	99.91	99.93	99.9	99.89	99.75	
1	Frequency	55	115	44	220	126	560
	Expected value	62.167	182.67	46.884	215.83	52.446	
	Percentage	0.01	0.02	0.01	0.04	0.02	0.11
	Row Percentage	9.82	20.54	7.86	39.29	22.5	
	Coloum Percentage	0.09	0.07	0.1	0.11	0.25	
Total	Frequency	58634	172290	44220	203568	49466	528178
	Percentage	11.1	32.62	8.37	38.54	9.37	100

OUTC * BP_1 Statistics for the table

Statistics	Degree of freedom	Value	Prob
Chi square	4	129.4459	<.0001
Likelihood ratio Chi square	4	103.9349	<.0001
Mantel-Haenszel Chi square	1	63.5947	<.0001
pi coefficient		0.0157	
Contingency coefficient		0.0157	
Kramer's V		0.0157	

Sample size = 528178

FREQ Procedure

Table OUTC * G1E_BMI_1

OUTC		G1E_BMI_1 0	1	2	3	4	Total
0	Frequency	12726	232821	119621	150728	11722	527618
	Expected value	12730	232834	119599	150726	11729	
	Percentage	2.41	44.08	22.65	28.54	2.22	99.89
	Row Percentage	2.41	44.13	22.67	28.57	2.22	
	Coloum Percentage	99.86	99.89	99.91	99.9	99.84	
1	Frequency	18	260	105	158	19	560
	Expected value	13.512	247.12	126.94	159.98	12.448	
	Percentage	0	0.05	0.02	0.03	0	0.11
	Row Percentage	3.21	46.43	18.75	28.21	3.39	
	Coloum Percentage	0.14	0.11	0.09	0.1	0.16	
Total	Frequency	12744	233081	119726	150886	11741	528178
	Percentage	2.41	44.13	22.67	28.57	2.22	100

OUTC * G1E_BMI_1 Statistics for the table

Statistics	Degree of freedom	Value	Prob
Chi square	4	9.4361	0.0511
Likelihood ratio Chi square	4	9.0392	0.0601
Mantel-Haenszel Chi square	1	0.2319	0.6301
pi coefficient		0.0042	
Contingency coefficient		0.0042	
Kramer's V		0.0042	

Sample size = 528178

FREQ Procedure

Table OUTC * G1E_FBS_1

OUTC		G1E_FBS_1 0	1	2	Total
0	Frequency	452552	63114	11952	527618
	Expected value	452541	63115	11962	
	Percentage	85.68	11.95	2.26	99.89
	Row Percentage	85.77	11.96	2.27	
	Coloum Percentage	99.9	99.89	99.81	
1	Frequency	469	68	23	560
	Expected value	480.31	66.989	12.696	
	Percentage	0.09	0.01	0	0.11
	Row Percentage	83.75	12.14	4.11	
	Coloum Percentage	0.1	0.11	0.19	
Total	Frequency	453021	63182	11975	528178
	Percentage	85.77	11.96	2.27	100

OUTC * G1E_FBS_1 Statistics for the table

Statistics	Degree of freedom	Value	Prob
Chi square	2	8.6526	0.0132
Likelihood ratio Chi square	2	7.0178	0.0299
Mantel-Haenszel Chi square	1	4.5629	0.0327
pi coefficient		0.004	
Contingency coefficient		0.004	
Kramer's V		0.004	

Sample size = 528178

FREQ Procedure

Table OUTC * G1E_TOT_CHOL_1

OUTC		G1E_TOT_CHOL_1 0	2	3	Total
0	Frequency	384097	114955	28566	527618
	Expected value	384088	114964	28566	
	Percentage	72.72	21.76	5.41	99.89
	Row Percentage	72.8	21.79	5.41	
	Coloum Percentage	99.9	99.89	99.9	
1	Frequency	399	131	30	560
	Expected value	407.66	122.02	30.319	
	Percentage	0.08	0.02	0.01	0.11
	Row Percentage	71.25	23.39	5.36	
	Coloum Percentage	0.1	0.11	0.1	
Total	Frequency	384496	115086	28596	528178
	Percentage	72.8	21.79	5.41	100

OUTC * G1E_TOT_CHOL_1 Statistics for the table

Statistics	Degree of freedom	Value	Prob
Chi square	2	0.8492	0.654
Likelihood ratio Chi square	2	0.8349	0.6587
Mantel-Haenszel Chi square	1	0.5163	0.4724
pi coefficient		0.0013	
Contingency coefficient		0.0013	
Kramer's V		0.0013	

Sample size = 528178

FREQ Procedure

Table OUTC * G1E_PHX_STK_1		G1E_PHX_STK_1		
		0	1	Total
OUTC				
0	Frequency	526078	1540	527618
	Expected value	526079	1539.4	
	Percentage	99.6	0.29	99.89
	Row Percentage	99.71	0.29	
	Coloum Percentage	99.89	99.94	
1	Frequency	559	1	560
	Expected value	558.37	1.6338	
	Percentage	0.11	0	0.11
	Row Percentage	99.82	0.18	
	Coloum Percentage	0.11	0.06	
Total	Frequency	526637	1541	528178
	Percentage	99.71	0.29	100

OUTC * G1E_PHX_STK_1 Statistics for the table

Statistics	Degree of freedom	Value	Prob
Chi square	1	0.2469	0.6193
Likelihood ratio Chi square	1	0.2868	0.5923
Continuity fix Chi square	1	0.011	0.9164
Mantel-Haenszel Chi square	1	0.2469	0.6193
pi coefficient		-0.0007	
Contingency coefficient		0.0007	
Kramer's V		-0.0007	

WARNING: 25%개의 cell이 5보다 적은 기대Frequency를 가지고 있습니다.
Chi square 검정은 올바르지 않을 수 있습니다.

Fisher's exact test	
(1,1) cell Frequency(F)	526078
Bottom side pValue Pr <= F	0.5137
Top side pValue Pr >= F	0.8055
Table Probability (P)	0.3191
Both side pValue Pr <= P	1

Odds ratio and Relative risk

Statistics	Value	95% CI	
Odds ratio	0.6111	0.0859	4.3487
Relative ratio(Coloum 1)	0.9989	0.9954	1.0024
Relative ratio(Coloum 2)	1.6345	0.2305	11.5906

Sample size = 528178

FREQ Procedure

Table OUTC * G1E_PHX_DM_1

OUTC		G1E_PHX_DM_1 0	G1E_PHX_DM_1 1	Total
0	Frequency	521271	6347	527618
	Expected value	521271	6347.3	
	Percentage	98.69	1.2	99.89
	Row Percentage	98.8	1.2	
	Coloum Percentage	99.89	99.89	
1	Frequency	553	7	560
	Expected value	553.26	6.7368	
	Percentage	0.1	0	0.11
	Row Percentage	98.75	1.25	
	Coloum Percentage	0.11	0.11	
Total	Frequency	521824	6354	528178
	Percentage	98.8	1.2	100

OUTC * G1E_PHX_DM_1 Statistics for the table

Statistics	Degree of freedom	Value	Prob
Chi square	1	0.0104	0.9187
Likelihood ratio Chi square	1	0.0103	0.9192
Continuity fix Chi square	1	0	1
Mantel-Haenszel Chi square	1	0.0104	0.9187
pi coefficient		0.0001	
Contingency coefficient		0.0001	
Kramer's V		0.0001	

Fisher's exact test	
(1,1) cell Frequency(F)	521271
Bottom side pValue Pr <= F	0.6381
Top side pValue Pr >= F	0.5111
Table Probability (P)	0.1493
Both side pValue Pr <= P	0.8451

Odds ratio and Relative risk

Statistics	Value	95% CI	
Odds ratio	1.0396	0.4931	2.1918
Relative ratio(Coloum 1)	1.0005	0.9912	1.0098
Relative ratio(Coloum 2)	0.9624	0.4607	2.0101

Sample size = 528178

FREQ Procedure

Table OUTC * G1E_PHX_HTDZ_1				
		G1E_PHX_HTDZ_1		
		0	1	Total
OUTC				
0	Frequency	525398	2220	527618
	Expected value	525399	2218.6	
	Percentage	99.47	0.42	99.89
	Row Percentage	99.58	0.42	
	Coloum Percentage	99.89	99.95	
1	Frequency	559	1	560
	Expected value	557.65	2.3548	
	Percentage	0.11	0	0.11
	Row Percentage	99.82	0.18	
	Coloum Percentage	0.11	0.05	
Total	Frequency	525957	2221	528178
	Percentage	99.58	0.42	100

OUTC * G1E_PHX_HTDZ_1 Statistics for the table

Statistics	Degree of freedom	Value	Prob
Chi square	1	0.7836	0.376
Likelihood ratio Chi square	1	1.0008	0.3171
Continuity fix Chi square	1	0.3119	0.5765
Mantel-Haenszel Chi square	1	0.7836	0.376
pi coefficient		-0.0012	
Contingency coefficient		0.0012	
Kramer's V		-0.0012	

WARNING: 25%개의 cell이 5보다 적은 기대Frequency를 가지고 있습니다.
Chi square 검정은 올바르지 않을 수 있습니다.

Fisher's exact test	
(1,1) cell Frequency(F)	525398
Bottom side pValue Pr <= F	0.3176
Top side pValue Pr >= F	0.9057
Table Probability (P)	0.2233
Both side pValue Pr <= P	0.7363

Odds ratio and Relative risk

Statistics	Value	95% CI	
Odds ratio	0.4234	0.0595	3.0122
Relative ratio(Coloum 1)	0.9976	0.9941	1.0011
Relative ratio(Coloum 2)	2.3563	0.3323	16.7053

Sample size = 528178

FREQ Procedure

Table OUTC * Q_FHX_HTN_1		
	Q_FHX_HTN_1	

		1	2	Total
OUTC				
0	Frequency	486864	40754	527618
	Expected value	486863	40755	
	Percentage	92.18	7.72	99.89
	Row Percentage	92.28	7.72	
	Coloum Percentage	99.89	99.89	
1	Frequency	516	44	560
	Expected value	516.74	43.256	
	Percentage	0.1	0.01	0.11
	Row Percentage	92.14	7.86	
	Coloum Percentage	0.11	0.11	
Total	Frequency	487380	40798	528178
	Percentage	92.28	7.72	100

OUTC * Q_FHX_HTN_1 Statistics for the table

Statistics	Degree of freedom	Value	Prob
Chi square	1	0.0139	0.9062
Likelihood ratio Chi square	1	0.0138	0.9065
Continuity fix Chi square	1	0.0015	0.9692
Mantel-Haenszel Chi square	1	0.0139	0.9062
pi coefficient		0.0002	
Contingency coefficient		0.0002	
Kramer's V		0.0002	

Fisher's exact test	
(1,1) cell Frequency(F)	486864
Bottom side pValue Pr <= F	0.5864
Top side pValue Pr >= F	0.4757
Table Probability (P)	0.0621
Both side pValue Pr <= P	0.8742

Odds ratio and Relative risk

Statistics	Value	95% CI	
Odds ratio	1.0187	0.7487	1.3861
Relative ratio(Coloum 1)	1.0014	0.9775	1.026
Relative ratio(Coloum 2)	0.9831	0.7402	1.3057

Sample size = 528178

FREQ Procedure

Table OUTC * Q_FHX_STK_1

OUTC		Q_FHX_STK_1 1	2	Total
0	Frequency	505991	21627	527618
	Expected value	505984	21634	
	Percentage	95.8	4.09	99.89
	Row Percentage	95.9	4.1	
	Coloum Percentage	99.9	99.86	
1	Frequency	530	30	560
	Expected value	537.04	22.962	
	Percentage	0.1	0.01	0.11
	Row Percentage	94.64	5.36	
	Coloum Percentage	0.1	0.14	
Total	Frequency	506521	21657	528178
	Percentage	95.9	4.1	100

OUTC * Q_FHX_STK_1 Statistics for the table

Statistics	Degree of freedom	Value	Prob
Chi square	1	2.252	0.1334
Likelihood ratio Chi square	1	2.0606	0.1512
Continuity fix Chi square	1	1.9434	0.1633
Mantel-Haenszel Chi square	1	2.252	0.1334
pi coefficient		0.0021	
Contingency coefficient		0.0021	
Kramer's V		0.0021	

Fisher's exact test	
(1,1) cell Frequency(F)	505991
Bottom side pValue Pr <= F	0.9411
Top side pValue Pr >= F	0.0856
Table Probability (P)	0.0267
Both side pValue Pr <= P	0.1348

Odds ratio and Relative risk

Statistics	Value	95% CI	
Odds ratio	1.3243	0.9165	1.9136
Relative ratio(Coloum 1)	1.0133	0.9935	1.0335
Relative ratio(Coloum 2)	0.7651	0.5401	1.084

Sample size = 528178

FREQ Procedure

Table OUTC * Q_FHX_DM_1					
		Q_FHX_DM_1		
		1	2	Total
OUTC				
0	Frequency	489974	37644	527618
	Expected value	489981	37637	
	Percentage	92.77	7.13	99.89
	Row Percentage	92.87	7.13	
	Coloum Percentage	99.89	99.91	
1	Frequency	527	33	560
	Expected value	520.05	39.947	
	Percentage	0.1	0.01	0.11
	Row Percentage	94.11	5.89	
	Coloum Percentage	0.11	0.09	
Total	Frequency	490501	37677	528178
	Percentage	92.87	7.13	100

OUTC * Q_FHX_DM_1 Statistics for the table

Statistics	Degree of freedom	Value	Prob
Chi square	1	1.3023	0.2538
Likelihood ratio Chi square	1	1.3787	0.2403
Continuity fix Chi square	1	1.1216	0.2896
Mantel-Haenszel Chi square	1	1.3023	0.2538
pi coefficient		-0.0016	
Contingency coefficient		0.0016	
Kramer's V		-0.0016	

Fisher's exact test	
(1,1) cell Frequency(F)	489974
Bottom side pValue Pr <= F	0.1439
Top side pValue Pr >= F	0.892
Table Probability (P)	0.0359
Both side pValue Pr <= P	0.2853

Odds ratio and Relative risk

Statistics	Value	95% CI	
Odds ratio	0.815	0.5733	1.1588
Relative ratio(Coloum 1)	0.9868	0.9665	1.0075
Relative ratio(Coloum 2)	1.2107	0.8695	1.686

Sample size = 528178

FREQ Procedure

Table OUTC * Q_DRK_FRQ_V0108_1

OUTC		Q_DRK_FRQ_V0108_1 1	2	3	4	5	Total
0	Frequency	175564	138166	163600	41397	8891	527618
	Expected value	175546	138129	163620	41416	8906.5	
	Percentage	33.24	26.16	30.97	7.84	1.68	99.89
	Row Percentage	33.27	26.19	31.01	7.85	1.69	
	Coloum Percentage	99.9	99.92	99.88	99.85	99.72	
1	Frequency	168	110	194	63	25	560
	Expected value	186.32	146.61	173.66	43.958	9.4532	
	Percentage	0.03	0.02	0.04	0.01	0	0.11
	Row Percentage	30	19.64	34.64	11.25	4.46	
	Coloum Percentage	0.1	0.08	0.12	0.15	0.28	
Total	Frequency	175732	138276	163794	41460	8916	528178
	Percentage	33.27	26.18	31.01	7.85	1.69	100

OUTC * Q_DRK_FRQ_V0108_1 Statistics for the table

Statistics	Degree of freedom	Value	Prob
Chi square	4	47.1909	<.0001
Likelihood ratio Chi square	4	39.0168	<.0001
Mantel-Haenszel Chi square	1	25.3261	<.0001
pi coefficient		0.0095	
Contingency coefficient		0.0095	
Kramer's V		0.0095	

Sample size = 528178

FREQ Procedure

Table OUTC * Q_DRK_AMT_V0108_1

OUTC		Q_DRK_AMT_V0108_1 0	1	2	3	4	Total
0	Frequency	177523	68642	173306	78734	29413	527618
	Expected value	177505	68634	173319	78732	29428	
	Percentage	33.61	13	32.81	14.91	5.57	99.89
	Row Percentage	33.65	13.01	32.85	14.92	5.57	
	Coloum Percentage	99.9	99.91	99.89	99.9	99.84	
1	Frequency	170	65	197	82	46	560
	Expected value	188.4	72.847	183.96	83.565	31.234	
	Percentage	0.03	0.01	0.04	0.02	0.01	0.11
	Row Percentage	30.36	11.61	35.18	14.64	8.21	
	Coloum Percentage	0.1	0.09	0.11	0.1	0.16	
Total	Frequency	177693	68707	173503	78816	29459	528178
	Percentage	33.64	13.01	32.85	14.92	5.58	100

OUTC * Q_DRK_AMT_V0108_1 Statistics for the table

Statistics	Degree of freedom	Value	Prob
Chi square	4	10.5882	0.0316
Likelihood ratio Chi square	4	9.7645	0.0446
Mantel-Haenszel Chi square	1	6.0644	0.0138
pi coefficient		0.0045	
Contingency coefficient		0.0045	
Kramer's V		0.0045	

Sample size = 528178

FREQ Procedure

Table OUTC * Q_SMK_YN_1

			Q_SMK_YN_1		
		1	2	3	Total
OUTC					
0	Frequency	224919	74617	228082	527618
	Expected value	224890	74607	228121	
	Percentage	42.58	14.13	43.18	99.89
	Row Percentage	42.63	14.14	43.23	
	Coloum Percentage	99.91	99.91	99.88	
1	Frequency	210	69	281	560
	Expected value	238.69	79.186	242.12	
	Percentage	0.04	0.01	0.05	0.11
	Row Percentage	37.5	12.32	50.18	
	Coloum Percentage	0.09	0.09	0.12	
Total	Frequency	225129	74686	228363	528178
	Percentage	42.62	14.14	43.24	100

OUTC * Q_SMK_YN_1 Statistics for the table

Statistics	Degree of freedom	Value	Prob
Chi square	2	11.0138	0.0041
Likelihood ratio Chi square	2	10.9114	0.0043
Mantel-Haenszel Chi square	1	9.5066	0.002
pi coefficient		0.0046	
Contingency coefficient		0.0046	
Kramer's V		0.0046	

Sample size = 528178

FREQ Procedure

Table OUTC * Q_SMK_NOW_AMT_V0108_1

OUTC		Q_SMK_NOW_AMT_V0108_1: 0	1	2	3	4	Total
0	Frequency	301120	40745	140626	43700	1427	527618
	Expected value	301083	40741	140635	43730	1429.5	
	Percentage	57.01	7.71	26.62	8.27	0.27	99.89
	Row Percentage	57.07	7.72	26.65	8.28	0.27	
	Coloum Percentage	99.91	99.9	99.89	99.83	99.72	
1	Frequency	283	39	158	76	4	560
	Expected value	319.56	43.241	149.27	46.413	1.5172	
	Percentage	0.05	0.01	0.03	0.01	0	0.11
	Row Percentage	50.54	6.96	28.21	13.57	0.71	
	Coloum Percentage	0.09	0.1	0.11	0.17	0.28	
Total	Frequency	301403	40784	140784	43776	1431	528178
	Percentage	57.06	7.72	26.65	8.29	0.27	100

OUTC * Q_SMK_NOW_AMT_V0108_1 Statistics for the table

Statistics	Degree of freedom	Value	Prob
Chi square	4	28.063	<.0001
Likelihood ratio Chi square	4	23.8885	<.0001
Mantel-Haenszel Chi square	1	19.0374	<.0001
pi coefficient		0.0073	
Contingency coefficient		0.0073	
Kramer's V		0.0073	

Sample size = 528178

FREQ Procedure

Table OUTC * Q_SMK_DRT_1

OUTC		Q_SMK_DRT_1: 0	1	2	3	4	5	Total
0	Frequency	238589	19831	58236	135647	55952	19363	527618
	Expected value	238562	19826	58217	135640	55987	19386	
	Percentage	45.17	3.75	11.03	25.68	10.59	3.67	99.89
	Row Percentage	45.22	3.76	11.04	25.71	10.6	3.67	
	Coloum Percentage	99.91	99.92	99.93	99.9	99.83	99.77	
1	Frequency	226	16	43	137	94	44	560
	Expected value	253.2	21.043	61.79	143.96	59.423	20.576	
	Percentage	0.04	0	0.01	0.03	0.02	0.01	0.11
	Row Percentage	40.36	2.86	7.68	24.46	16.79	7.86	
	Coloum Percentage	0.09	0.08	0.07	0.1	0.17	0.23	
Total	Frequency	238815	19847	58279	135784	56046	19407	528178
	Percentage	45.21	3.76	11.03	25.71	10.61	3.67	100

OUTC * Q_SMK_DRT_1 Statistics for the table

Statistics	Degree of freedom	Value	Prob
Chi square	5	57.028	<.0001
Likelihood ratio Chi square	5	48.2592	<.0001
Mantel-Haenszel Chi square	1	24.1417	<.0001
pi coefficient		0.0104	
Contingency coefficient		0.0104	
Kramer's V		0.0104	

Sample size = 528178

FREQ Procedure

Table OUTC * Q_PA_FRQ_1

OUTC		Q_PA_FRQ_1 1	2	3	4	5	Total
0	Frequency	229395	200536	68982	12875	15830	527618
	Expected value	229406	200535	68973	12871	15833	
	Percentage	43.43	37.97	13.06	2.44	3	99.89
	Row Percentage	43.48	38.01	13.07	2.44	3	
	Coloum Percentage	99.89	99.89	99.91	99.92	99.87	
1	Frequency	254	212	64	10	20	560
	Expected value	243.49	212.84	73.206	13.661	16.805	
	Percentage	0.05	0.04	0.01	0	0	0.11
	Row Percentage	45.36	37.86	11.43	1.79	3.57	
	Coloum Percentage	0.11	0.11	0.09	0.08	0.13	
Total	Frequency	229649	200748	69046	12885	15850	528178
	Percentage	43.48	38.01	13.07	2.44	3	100

OUTC * Q_PA_FRQ_1 Statistics for the table

Statistics	Degree of freedom	Value	Prob
Chi square	4	3.2072	0.5238
Likelihood ratio Chi square	4	3.3192	0.5059
Mantel-Haenszel Chi square	1	0.6015	0.438
pi coefficient		0.0025	
Contingency coefficient		0.0025	
Kramer's V		0.0025	

Sample size = 528178

FREQ Procedure

Table OUTC * SMK_1

OUTC		SMK_1 = 0	SMK_1 = 1	Total
0	Frequency	299536	228082	527618
	Expected value	299497	228121	
	Percentage	56.71	43.18	99.89
	Row Percentage	56.77	43.23	
	Coloum Percentage	99.91	99.88	
1	Frequency	279	281	560
	Expected value	317.88	242.12	
	Percentage	0.05	0.05	0.11
	Row Percentage	49.82	50.18	
	Coloum Percentage	0.09	0.12	
Total	Frequency	299815	228363	528178
	Percentage	56.76	43.24	100

OUTC * SMK_1 Statistics for the table

Statistics	Degree of freedom	Value	Prob
Chi square	1	11.0096	0.0009
Likelihood ratio Chi square	1	10.9066	0.001
Continuity fix Chi square	1	10.7283	0.0011
Mantel-Haenszel Chi square	1	11.0096	0.0009
pi coefficient		0.0046	
Contingency coefficient		0.0046	
Kramer's V		0.0046	

Fisher's exact test

(1,1) cell Frequency(F)	299536
Bottom side pValue Pr <= F	0.9996
Top side pValue Pr >= F	0.0006
Table Probability (P)	0.0001
Both side pValue Pr <= P	0.001

Odds ratio and Relative risk

Statistics	Value	95% CI	
Odds ratio	1.3227	1.1207	1.5611
Relative ratio(Coloum 1)	1.1395	1.0486	1.2383
Relative ratio(Coloum 2)	0.8615	0.7932	0.9357

Sample size = 528178
