# Supplementary material for: Optimal target blood pressure for the primary prevention of hemorrhagic stroke: a nationwide observational study
Source: Front Neurol. 2023 Oct 9;14:1268542. doi: 10.3389/fneur.2023.1268542 (PMC10593468; doi:10.3389/fneur.2023.1268542)
Supplement: Supplementary file 2 [file Data_Sheet_2.PDF]

The PHREG Procedure

Model Information	
Data Set	SNUBH.G1_CTR_MALEHSTK_HTNMD0
Dependent Variable	TIME
Censoring Variable	OUTC
Censoring Value(s)	0
Ties Handling	BRESLOW

Number of Observations Read	528178
Number of Observations Used	528178

Class Level Information						
Class	Value	Design Variables				
AGE_1	0	0	0	0		
	1	1	0	0		
	2	0	1	0		
	3	0	0	1		
BP_1	0	0	0	0	0	
	1	1	0	0	0	
	2	0	1	0	0	
	3	0	0	1	0	
	4	0	0	0	1	
G1E_BMI_1	0	0	0	0	0	
	1	1	0	0	0	
	2	0	1	0	0	
	3	0	0	1	0	
	4	0	0	0	1	
G1E_FBS_1	0	0	0			
	1	1	0			
	2	0	1			
Q_DRK_FRQ_V0108_1	1	0	0	0	0	
	2	1	0	0	0	
	3	0	1	0	0	
	4	0	0	1	0	
	5	0	0	0	1	
SMK_1	0	0				
	1	1				

Summary of the Number of Event and Censored Values			
Total	Event	Censored	Percent Censored
528178	560	527618	99.89

The PHREG Procedure

Convergence Status
Convergence criterion (GCONV=1E-8) satisfied.

Model Fit Statistics

Criterion	Without Covariates	With Covariates
-2 LOG L	14757.918	14511.887
AIC	14757.918	14547.887
SBC	14757.918	14625.790

Testing Global Null Hypothesis: BETA=0

Test	Chi-Square	DF	Pr > ChiSq
Likelihood Ratio	246.0305	18	<.0001
Score	299.2670	18	<.0001
Wald	273.6749	18	<.0001

Type 3 Tests

Effect	DF	Wald Chi-Square	Pr > ChiSq
AGE_1	3	94.3005	<.0001
BP_1	4	77.2543	<.0001
G1E_BMI_1	4	17.8812	0.0013
G1E_FBS_1	2	6.0552	0.0484
Q_DRK_FRQ_V0108_1	4	14.2880	0.0064
SMK_1	1	13.1438	0.0003

Analysis of Maximum Likelihood Estimates

Parameter		DF	Parameter Estimate	Standard Error	Chi-Square	Pr > ChiSq	Hazard Ratio	95% Hazard Ratio Confidence Limits		Label
AGE_1	1	1	0.56206	0.10228	30.2002	<.0001	1.754	1.436	2.144	AGE_1 1
AGE_1	2	1	0.91943	0.11982	58.8831	<.0001	2.508	1.983	3.172	AGE_1 2
AGE_1	3	1	1.40775	0.18272	59.3613	<.0001	4.087	2.857	5.847	AGE_1 3
BP_1	1	1	-0.18623	0.16531	1.2691	0.2599	0.830	0.600	1.148	BP_1 1
BP_1	2	1	0.23353	0.20453	1.3037	0.2535	1.263	0.846	1.886	BP_1 2
BP_1	3	1	0.28207	0.15526	3.3005	0.0693	1.326	0.978	1.797	BP_1 3
BP_1	4	1	0.99298	0.17221	33.2493	<.0001	2.699	1.926	3.783	BP_1 4
G1E_BMI_1	1	1	-0.30960	0.24504	1.5964	0.2064	0.734	0.454	1.186	G1E_BMI_1 1
G1E_BMI_1	2	1	-0.68214	0.25866	6.9549	0.0084	0.506	0.304	0.839	G1E_BMI_1 2
G1E_BMI_1	3	1	-0.60618	0.25442	5.6765	0.0172	0.545	0.331	0.898	G1E_BMI_1 3
G1E_BMI_1	4	1	-0.23083	0.33665	0.4701	0.4929	0.794	0.410	1.536	G1E_BMI_1 4
G1E_FBS_1	1	1	-0.32689	0.13364	5.9827	0.0144	0.721	0.555	0.937	G1E_FBS_1 1
G1E_FBS_1	2	1	-0.00747	0.21878	0.0012	0.9728	0.993	0.646	1.524	G1E_FBS_1 2

The PHREG Procedure

Analysis of Maximum Likelihood Estimates

Parameter		DF	Parameter Estimate	Standard Error	Chi-Square	Pr > ChiSq	Hazard Ratio	95% Hazard Ratio Confidence Limits		Label
Q_DRK_FRQ_V0108_1	2	1	-0.15080	0.12517	1.4513	0.2283	0.860	0.673	1.099	Q_DRK_FRQ_V0108_1 2
Q_DRK_FRQ_V0108_1	3	1	0.14021	0.11098	1.5961	0.2065	1.151	0.926	1.430	Q_DRK_FRQ_V0108_1 3
Q_DRK_FRQ_V0108_1	4	1	0.22683	0.15387	2.1730	0.1405	1.255	0.928	1.696	Q_DRK_FRQ_V0108_1 4
Q_DRK_FRQ_V0108_1	5	1	0.58434	0.21998	7.0561	0.0079	1.794	1.166	2.761	Q_DRK_FRQ_V0108_1 5
SMK_1	1	1	0.32782	0.09042	13.1438	0.0003	1.388	1.163	1.657	SMK_1 1
