# Supplementary material for: Optimal target blood pressure for the primary prevention of hemorrhagic stroke: a nationwide observational study
Source: Front Neurol. 2023 Oct 9;14:1268542. doi: 10.3389/fneur.2023.1268542 (PMC10593468; doi:10.3389/fneur.2023.1268542)
Supplement: Supplementary file 3 [file Data_Sheet_3.PDF]

The PHREG Procedure

Model Information	
Data Set	SNUBH.G1_CTR_FEMHSTK_HTNMD0
Dependent Variable	TIME
Censoring Variable	OUTC
Censoring Value(s)	0
Ties Handling	BRESLOW

Number of Observations Read	419200
Number of Observations Used	419200

Class Level Information					
Class	Value	Design Variables			
AGE_1	0	0	0	0	
	1	1	0	0	
	2	0	1	0	
	3	0	0	1	
BP_1	0	0	0	0	0
	1	1	0	0	0
	2	0	1	0	0
	3	0	0	1	0
	4	0	0	0	1
G1E_BMI_1	0	0	0	0	0
	1	1	0	0	0
	2	0	1	0	0
	3	0	0	1	0
	4	0	0	0	1
G1E_FBS_1	0	0	0		
	1	1	0		
	2	0	1		
SMK_1	0	0			
	1	1			
Q_PA_FRQ_1	1	0	0	0	0
	2	1	0	0	0
	3	0	1	0	0
	4	0	0	1	0
	5	0	0	0	1

Summary of the Number of Event and Censored Values			
Total	Event	Censored	Percent Censored
419200	353	418847	99.92

The PHREG Procedure

Convergence Status
Convergence criterion (GCONV=1E-8) satisfied.

Model Fit Statistics		
Criterion	Without Covariates	With Covariates
-2 LOG L	9139.683	8931.297
AIC	9139.683	8967.297
SBC	9139.683	9036.893

Testing Global Null Hypothesis: BETA=0			
Test	Chi-Square	DF	Pr > ChiSq
Likelihood Ratio	208.3860	18	<.0001
Score	238.4856	18	<.0001
Wald	204.0037	18	<.0001

Type 3 Tests			
Effect	DF	Wald Chi-Square	Pr > ChiSq
AGE_1	3	82.7355	<.0001
BP_1	4	47.7552	<.0001
G1E_BMI_1	4	4.3415	0.3618
G1E_FBS_1	2	1.4446	0.4856
SMK_1	1	12.4586	0.0004
Q_PA_FRQ_1	4	3.6032	0.4624

Analysis of Maximum Likelihood Estimates										
Parameter		DF	Parameter Estimate	Standard Error	Chi-Square	Pr > ChiSq	Hazard Ratio	95% Hazard Ratio Confidence Limits		Label
AGE_1	1	1	1.13223	0.13504	70.3007	<.0001	3.103	2.381	4.043	AGE_1 1
AGE_1	2	1	1.08662	0.17211	39.8590	<.0001	2.964	2.115	4.154	AGE_1 2
AGE_1	3	1	1.55464	0.24407	40.5712	<.0001	4.733	2.934	7.637	AGE_1 3
BP_1	1	1	0.24272	0.13917	3.0415	0.0812	1.275	0.970	1.674	BP_1 1
BP_1	2	1	0.27425	0.25265	1.1783	0.2777	1.316	0.802	2.159	BP_1 2
BP_1	3	1	0.91267	0.14946	37.2895	<.0001	2.491	1.858	3.339	BP_1 3
BP_1	4	1	1.05618	0.22469	22.0957	<.0001	2.875	1.851	4.466	BP_1 4
G1E_BMI_1	1	1	0.21543	0.25712	0.7020	0.4021	1.240	0.749	2.053	G1E_BMI_1 1
G1E_BMI_1	2	1	0.26591	0.28671	0.8602	0.3537	1.305	0.744	2.288	G1E_BMI_1 2
G1E_BMI_1	3	1	0.12005	0.29473	0.1659	0.6838	1.128	0.633	2.009	G1E_BMI_1 3
G1E_BMI_1	4	1	-1.55297	1.03573	2.2482	0.1338	0.212	0.028	1.611	G1E_BMI_1 4
G1E_FBS_1	1	1	-0.27017	0.24064	1.2605	0.2616	0.763	0.476	1.223	G1E_FBS_1 1
G1E_FBS_1	2	1	0.16579	0.45613	0.1321	0.7162	1.180	0.483	2.886	G1E_FBS_1 2

The PHREG Procedure

Analysis of Maximum Likelihood Estimates										
Parameter		DF	Parameter Estimate	Standard Error	Chi-Square	Pr > ChiSq	Hazard Ratio	95% Hazard Ratio Confidence Limits		Label
SMK_1	1	1	0.96800	0.27425	12.4586	0.0004	2.633	1.538	4.506	SMK_1 1
Q_PA_FRQ_1	2	1	-0.08375	0.13324	0.3951	0.5296	0.920	0.708	1.194	Q_PA_FRQ_1 2
Q_PA_FRQ_1	3	1	0.03246	0.17401	0.0348	0.8520	1.033	0.734	1.453	Q_PA_FRQ_1 3
Q_PA_FRQ_1	4	1	0.36149	0.28711	1.5852	0.2080	1.435	0.818	2.520	Q_PA_FRQ_1 4
Q_PA_FRQ_1	5	1	-0.41206	0.36219	1.2944	0.2552	0.662	0.326	1.347	Q_PA_FRQ_1 5
